# NF-κB: A Double-Edged Sword Controlling Inflammation

**DOI:** 10.3390/biomedicines10061250

**Published:** 2022-05-27

**Authors:** Danhui Liu, Zhenyu Zhong, Michael Karin

**Affiliations:** 1 Department of Immunology, University of Texas Southwestern Medical Center, Dallas, TX 75390, USA; danhui.liu@utsouthwestern.edu; 2 Laboratory of Gene Regulation and Signal Transduction, Department of Pharmacology, University of California San Diego, La Jolla, CA 92093, USA

**Keywords:** NF-κB, inflammation, NLRP3 inflammasome, mitochondrial damage, mitochondrial DNA, mitophagy, cancer

## Abstract

Inflammation, when properly mounted and precisely calibrated, is a beneficial process that enables the rapid removal of invading pathogens and/or cellular corpses and promotes tissue repair/regeneration to restore homeostasis after injury. Being a paradigm of a rapid response transcription factor, the nuclear factor-kappa B (NF-κB) transcription factor family plays a central role in amplifying inflammation by inducing the expression of inflammatory cytokines and chemokines. Additionally, NF-κB also induces the expression of pro-survival and -proliferative genes responsible for promoting tissue repair and regeneration. Paradoxically, recent studies have suggested that the NF-κB pathway can also exert inhibitory effects on pro-inflammatory cytokine production to temper inflammation. Here, we review our current understanding about the pro- and anti-inflammatory roles of NF-κB and discuss the implication of its dichotomous inflammation-modulating activity in the context of inflammasome activation and tumorigenesis.

## 1. Introduction

Inflammation is an essential innate immune process exploited by the host to initiate protective responses against various insults. Upon pathogen invasion and tissue damage, a rapidly mounted inflammatory response is critical for neutralizing/eliminating pathogens and/or cellular corpses [1]. However, once this goal is achieved, the inflammatory flame needs to be extinguished promptly to initiate tissue repair and regeneration, which ultimately restores homeostasis and organismal health [2]. When the host fails to resolve inflammation, as evidenced by prolonged, uncontrolled immune activation even after the clearance of insults, it often results in an impediment of tissue repair, leading to the loss of normal tissue function and the consequent development of chronic syndromes such as autoinflammatory/autoimmune diseases, degenerative or metabolic disorders, and various types of cancer [2]. Therefore, a well-balanced and precisely controlled inflammatory response is crucial for insult clearance and tissue repair, while avoiding devastating immunopathology—ultimately restoring homeostasis [1,3,4,5].

Inflammatory responses are initiated upon host recognition of inflammatory cues in the form of either pathogen-derived molecules (PAMPs) or self-danger signals generated during tissue damage (DAMPs), by diverse pattern recognition receptors (PRR) [5,6]. NOD-like receptor family pyrin domain containing 3 (NLRP3) is a PRR that acts as a dominant innate immune sensor for tissue damage, and thus plays an indispensable role in igniting sterile inflammation [1,7]. By sensing self-danger signals, NLRP3 undergoes a conformational change that results in unfolding and binding to the adaptor protein ASC through homotypic pyrin–pyrin domain interactions, ultimately leading to ASC nucleation. The ASC scaffold then recruits the effector pro-caspase-1 to eventually form a large cytosolic protein complex termed the NLRP3 inflammasome, whose outcome is self-cleavage and autoactivation of pro-caspase-1, generating mature caspase-1. Activated caspase-1 then processes pro-IL-1β and pro-IL-18 into their bioactive forms, respectively, to initiate inflammation [6]. NLRP3 inflammasome activation is crucial for mounting protective immunity in response to injury by stimulating punchy damage clearance and tissue repair pathways [8]. However, aberrant NLRP3 inflammasome activation has also been shown to drive the progression of many major human diseases, including various types of cancer, as well as metabolic and degenerative disorders [9,10,11].

Nuclear factor-κB (NF-κB) was first discovered in 1986 by David Baltimore’s group as a transcription factor involved in B-cell development and activation [12,13,14]. Subsequent studies established a broad role of this transcription factor in diverse cellular processes, including inflammation, cell proliferation and survival, differentiation of effector and regulatory T cells, and maturation of dendritic cells. Acting as a perfect example of a rapid response transcription factor, NF-κB family members are retained in the cytoplasm in an inactive state in resting cells by binding to the inhibitor of NF-κB (IκB) [13]. Upon stimulation by PAMPs, DAMPs, or proinflammatory cytokines, the engagement of PRR and cytokine receptors triggers downstream signaling cascades, leading to the activation of the IκB kinase (IKK) complex. IKK then phosphorylates and promotes proteasomal degradation of the IκBs to liberate NF-κB dimers for nuclear translocation, resulting in the expression of pro-survival and -proliferative genes, as well as various cytokines and chemokines to propagate inflammation. Once the inflammatory insults are cleared and inflammation is no longer needed, IKK is deactivated and IκBs accumulate and remove NF-κB dimers from the DNA back to the cytoplasm [15,16].

As a result of its key role in initiating an inflammatory response, NF-κB was thought to be an “ideal” drug target for the treatment of diverse inflammatory diseases [17]. However, quite unexpectedly, the pharmacologic or genetic inhibition of NF-κB was found to exacerbate, rather than attenuate, inflammation in many preclinical disease models, which was then recapitulated in several large clinical trials. This led to the termination of several drug-development programs targeting IKK or other components of the NF-κB pathway [1,3,17,18]. It was not clear until recently that these seemingly counterintuitive results can at least partially be explained by the fact that NF-κB also acts as a macrophage-intrinsic negative regulator of the NLRP3 inflammasome. In this review, we summarize recent advancements in understanding the pro- and anti-inflammatory properties of NF-κB and discuss its implication in inflammasome activation and tumorigenesis.

## 2. The NF-κB Signaling Pathway

The NF-κB family transcription factors consist of five different DNA binding proteins that share a Rel homology region (cRel, RelA, RelB, NF-κB1, and NF-κB2) and can form up to 15 homodimers and heterodimers [19,20]. In resting cells, these dimers are kept in the cytoplasm in an inactive form through binding to IκB proteins that mask their nuclear localization sequence (NLS).

There are two distinct NF-κB signaling pathways operating in the cell: canonical and alternative pathways. The canonical pathway entails rapid and transient activation in response to stimulation with PAMPs/DAMPs (e.g., LPS, polyI:C, and CpG DNA) and proinflammatory cytokines (e.g., TNF and IL-1), or upon T- and B-cell receptor engagement [21]. Although the upstream signaling events vary among different NF-κB activating receptors, the downstream signaling converges on the IKK complex comprised of the catalytic subunits IKKα/β and the regulatory subunit IKKγ/NEMO. Once activated, the IKK complex phosphorylates IκB molecules on two adjacent serine residues, thereby promoting K48-linked ubiquitination to induce the proteasomal degradation of IκBs. The liberated NF-κB dimers then translocate into the nucleus where they initiate the transcription of a large set of genes [13], including pro-inflammatory cytokines and pro-survival molecules, as well as enzymes that generate non-protein inflammatory mediators, so as to amplify inflammatory responses and/or promote cell proliferation and survival [14]. Given its indispensable roles in regulating multiple cellular functions, the NF-κB pathway needs to be tightly regulated to avoid excessive activation that would otherwise cause pathology. Indeed, the NF-κB pathway can be negatively regulated at multiple levels. *Nfkbia*, which encodes IκBα, is one of the NF-κB target genes that serves as an inhibitor by binding to the NF-κB dimer, thereby terminating the NF-κB transcriptional activity. Apart from this, deubiquitylation also negatively regulates NF-κB activity. For example, CYLD (cylindromatosis), a deubiquitinase, was shown to negatively regulate the canonical NF-κB pathway by disassembling K63-ubiquitin chains on TRAF2, TRAF6, and NEMO, thereby inhibiting IKK activation [22].

The alternative NF-κB pathway plays an essential role in inducing genes associated with secondary lymphoid organ development and maintenance [14]. However, in contrast to the rapidly induced canonical NF-κB pathway, activation of the alternative pathway requires *de novo* synthesis of NF-κB-inducing kinase (NIK, also known as MAP3K14), and therefore has slow kinetics. TNF superfamily cytokines, including lymphotoxin (LT), receptor activator of NF-κB ligand (RANKL; also known as TNFSF11), CD40 ligand (CD40L), and B-cell activating factor of the TNF family (BAFF; also known as TNFSF13B), serve as the ligands for the alternative NF-κB pathway [23,24,25], which, through activating IKKα homodimers, drive the NF-κB2/p52-RELB dimer activation [25,26].

## 3. Pro- and Anti-Inflammatory Properties of NF-κB

NF-κB is traditionally viewed as a key transcriptional activator of an arsenal of pro-inflammatory, -survival, and -proliferative molecules [17]. Consistent with this notion, it is well-documented that most of the pro-inflammatory cytokine/chemokine genes possess NF-κB-binding site(s) in their promoter/enhancer regions, and the activation of NF-κB is essential for their induction in response to a large array of immunostimulatory stimuli [27]. Moreover, overactivation of NF-κB signaling is evident in many chronic inflammatory disorders, such as inflammatory bowel disease (IBD) [28,29], rheumatoid arthritis (RA) [30], and psoriasis, among others [28,30,31,32,33] (Table 1). The ability of NF-κB to induce TNF expression is thought to be a major pathogenic driver of these diseases. Consistently, all of these disorders have responded to anti-TNF therapy and NF-κB inhibitors [34,35,36,37,38,39,40]. In acute inflammatory conditions such as sepsis, genetic polymorphisms potentiating NF-κB activation have been found to increase mortality because of excessive inflammation [41,42]. Together, these findings imply that targeting NF-κB signaling might be beneficial for treating inflammatory diseases [17]. However, contradicting this notion, pharmacological or genetic inhibition of NF-κB has been shown to exacerbate, rather than attenuate, inflammation under various disease settings [3,18], leading to the termination of several drug development pipelines aiming to inhibit IKK-driven NF-κB activation to eliminate inflammation.

This unexpected anti-inflammatory property of NF-κB can be both indirect and direct [1,3,17]. The former often takes place at barrier surfaces (e.g., skin and intestine), where NF-κB-mediated pro-survival signaling ensures proper barrier function to prevent microbial translocation [29,43,44]. In addition to classical pro-survival molecules, including BCL-XL, FLICE-like inhibitory protein (FLIP), and members of the inhibitor of apoptosis (IAP) family [16], the expression of other molecules involved in the preservation of epithelial integrity is also under the control of NF-κB [29]. In line with this concept, the loss of IKKβ in intestinal epithelial cells (IECs) drastically increases susceptibility to chemical-induced colitis in mice [43]. Similarly, mice lacking IKKγ/NEMO in IECs display a severe and spontaneous inflammatory condition [44], and the absence of IKKγ in mouse keratinocytes can also lead to the development of a psoriasis-like inflammatory disease [29]. In contrast to these indirect effects, the direct anti-inflammatory function of NF-κB is largely attributed to its ability to limit the production of a key proinflammatory cytokine—IL-1β. This was first demonstrated in our earlier study in which pharmacologic or genetic inhibition of NF-κB unexpectedly exacerbated IL-1β-dependent inflammation in vivo [18]. Mice lacking Ikkβ expression in myeloid cells were more susceptible to lipopolysaccharide (LPS)- or bacteria-induced septic shock. Similar results were observed after repetitively treating WT mice with a specific IKKβ inhibitor. Moreover, spontaneous development of progressive neutrophilia was observed in mice genetically or pharmaceutically deprived of IKKβ in myeloid cells owing to the dramatically augmented IL-1β production [45]. These results indicate that, in addition to promoting pro-IL-1β expression, NF-κB functions to limit the production of bioactive IL-1β—a process mediated by inflammasome assembly and subsequent caspase-1 activation.

**Table 1:** Pro- and anti-inflammatory properties of NF-κB in inflammatory diseases.

Type of Diseases (Models)	Role and Mechanism of Action	References
Pro-inflammatory role		
IBD	NF-κB p65 is potently activated in TNBS-induced experimental colitis and local p65 inhibition abrogates clinical and histological signs of colitis.	[34]
	Blockade of NF-κB attenuates TNBS-induced chronic inflammation associated intestinal fibrosis in mice.	[46]
	Blocking RhoA/Rho-kinase pathway prevents experimental colitis via NF-κB inhibition.	[47]
RA	IKKβ overexpression in the joints of rats results in significant synovial inflammation. Intraarticular transfer of IKKβ-dominant negative adenoviral constructs decreases NF-κB expression in the joints and ameliorates the severity of arthritis.	[30,38]
	Gene polymorphism of NF-κB pathway components exists in patients with autoimmune rheumatic disease.	[48,49,50]
Skin inflammation	Constitutively active NF-κB/RelA is present in uninvolved epidermis from psoriasis patients, and etanercept treatment significantly downregulates phosphorylated NF-κB/RelA correlating with the restoration of normal markers of keratinocyte differentiation and clinical outcome.	[31,32,33]
Sepsis	Increased NF-κB binding activity is present after the injection of LPS in mice. Intravenous somatic gene transfer with IκBα given before LPS attenuates renal NF-κB binding activity and increases survival.	[41,42]
**Anti-inflammatory role**		
IBD	Ikkβ depletion in IECs increases colonic inflammation in a DSS-induced mice model of colitis.	[43]
	IECs’ specific inhibition of NEMO induces apoptosis of colonic epithelial cells, resulting in the disruption of epithelial integrity and intestinal immune homeostasis, thereby causing severe chronic intestinal inflammation in mice.	[29,44,51]
Skin inflammation	Inhibition of NF-κB in the mouse epidermis disturbs skin homeostasis and triggers TNF-dependent skin inflammation, epidermal hyperplasia, and subsequent development of squamous cell carcinoma.	[29,52]
Endotoxin-induced infection	Mice with a targeted IKKβ deletion in myeloid cells are more susceptible to endotoxin-induced shock owing to overwhelmed IL-1β production.	[18]
	Mice deprived of IKKβ in monocytes develop a spontaneous neutrophilia owing to augmented IL-1β production.	[14,45,53]

IBD: inflammatory bowel disease; TNBS: 2,4,6-trinitrobenzene sulfonic acid; RA: rheumatoid arthritis; LPS: lipopolysaccharide; IECs: intestinal epithelial cells; DSS: dextran sodium sulfate; NEMO: NF-κB essential modulator.

## 4. NF-κB in NLRP3 Inflammasome Activation

Inflammasomes, a group of multi-protein signaling platforms, are key mediators of innate immunity and play indispensable roles in the initiation and propagation of inflammation [54]. The NLRP3 inflammasome, the most extensively studied member in this group, is a key immune sensor of tissue damage [3,55]. A “two-step” process, namely “priming” and “activation”, is required for NLRP3 inflammasome assembly [3,6,56]. Priming entails the detection of DAMPs or PAMPs by PRRs to drive NF-κB-dependent *de novo* synthesis of pro-IL-1β and upregulation of NLRP3. In contrast, activation takes place after cell exposure to chemically and structurally diverse NLRP3 activators, including ATP, pathogen-derived factors, and many microparticle-shaped insults [1,7], triggering the assembly of the NLRP3 inflammasome complex and subsequent self-cleavage and autoactivation of caspase-1, which in turn processes pro-IL-1β/pro-IL-18 into their mature forms to ignite inflammation. Additionally, active caspase-1 also cleaves Gasdermin D (GSDMD), whose N-terminal fragments insert into the plasma membrane and form pores to facilitate the release of IL-1β/IL-18, as well as to initiate an inflammatory form of cell death, named pyroptosis [57,58].

Inflammasome priming is a multifaceted process involving the transcriptional induction of inflammasome components followed by a series of posttranslational modifications necessary for their subsequent activation [59,60]. Priming starts with NF-κB-dependent transcription of cytokine precursors (e.g., pro-IL-1β) and upregulation of NLRP3 itself [61,62]. Ligands for PRRs or cytokine receptors, such as LPS and TNF, serve as priming stimuli that activate NF-κB signaling. Upregulation of NLRP3 increases its abundance above a threshold, allowing for subsequent inflammasome assembly when cells encounter NLRP3 activators [59,63,64]. Moreover, recent studies have also revealed transcription-independent priming events achieved through the posttranslational modifications (PTMs) of inflammasome components [59]. For instance, 10 min acute priming with LPS enhances NLRP3 inflammasome activation in the absence of NLRP3 upregulation [65,66], and the simultaneous addition of priming and activation stimuli can also activate NLRP3 [65,66,67,68,69]. The adapter molecule MyD88 and the IL-1 receptor-associated kinases IRAK-1 and IRAK-4 are essential for triggering multiple PTMs processes, including the deubiquitylation and phosphorylation of NLRP3, thereby contributing to this rapid priming event [62,68,69,70]. 

The chemical and structural diversity of NLRP3 inflammasome activators suggests that they need to operate through a common downstream signaling intermediate [3,8]. Numerous independent studies have collectively demonstrated that mitochondrial damage is a common signaling event downstream of all NLRP3 activators, resulting in the production of oxidized mitochondrial DNA (ox-mtDNA) that binds to and activates NLRP3 [3,71]. The concept that mitochondria are a signaling hub that controls NLRP3 inflammasome activation was initially proposed by the late Jurg Tschopp and Augustine Choi [72,73], who independently demonstrated that NLRP3 activator-induced mitochondrial damage is indispensable for NLRP3 inflammasome activation. Shortly thereafter, Moshe Arditi’s group further extended our understanding by showing that ox-mtDNA released from the damaged mitochondria in apoptotic cells serves as an endogenous activator that binds to and activates NLRP3 [71].

As a dominant immune sensor of tissue damage that ignites inflammation in response to a breach of homeostasis, the NLRP3 inflammasome activity must be precisely tuned and tightly controlled to avoid immunopathology [3,8]. Therefore, keeping mitochondrial damage under control is vital for preventing NLRP3 inflammasome overactivation [74,75,76]. In macrophages, this mission is mainly carried out by the autophagy machinery, which, through selective clearance of the damaged mitochondria via mitophagy, acts as a “brake” to restrict excessive NLRP3 inflammasome activation [56,72,73,77]. The first evidence that autophagy may have an inhibitory role for NLRP3 inflammasome came from Shizuo Akira’s group, who showed that Atg16L1 deficiency in mice resulted in IL-1β overproduction by macrophages [77]. In support of this finding, the Tschopp group and Choi group later revealed that autophagy-mediated clearance of damaged mitochondria inhibits NLRP3 inflammasome activation, thereby restricting excessive IL-1β production [72,73]. 

**Figure 1:**
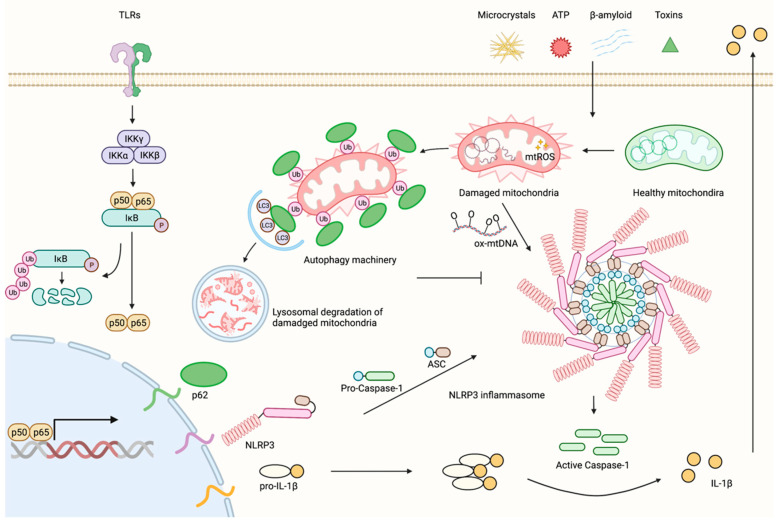
NF-κB controls both the gas pedal and brake of the NLRP3 inflammasome. During inflammasome priming, TLR activation results in NF-κB-dependent robust transcriptional upregulation of NLRP3 and *de novo* synthesis of pro-IL-1β. In parallel, NF-κB activation also induces p62 upregulation, although with slower kinetics. In the inflammasome activation step, various NLRP3 inflammasome activators trigger mitochondrial damage, resulting in the generation of oxidized mtDNA (ox-mtDNA), which is subsequently released from the damaged mitochondria to the cytosol, where it binds to and activates NLRP3. This leads to the assembly of the inflammasome complex, followed by autocleavage and activation of caspase-1, which in turn proteolytically processes pro-IL-1β into its mature and bioactive form, thereby igniting inflammation. Meanwhile, to prevent NLRP3 overactivation, p62 induced upon NF-κB activation promotes the autophagic degradation of the damaged mitochondria, a process also known as mitophagy, and thereby restricts NLRP3 hyperactivation. In summary, NF-κB controls both the gas pedal and brake of NLRP3 inflammasome to generate a well-balanced immune response that focuses on the removal of insults and tissue repair while avoiding immunopathology (the figure was created using BioRender).

To gain further mechanistic insights into the negative regulatory network that keeps NLRP3 inflammasome activity in check in macrophages, we recently discovered that NF-κB is a driver for this circuit by inducing the expression of an autophagy adaptor molecule, called p62 [3], also known as sequestosome 1 (SQSTM1). p62 functions to bridge autophagy machinery with its cargo (e.g., protein complexes and damaged mitochondria), thereby targeting the cargo for lysosomal degradation [57,78]. We found that the expression of p62 is strongly induced, albeit with delayed kinetics relative to pro-IL-1β, during priming, to prepare the macrophage for efficient clearance of the damaged mitochondria at the inflammasome activation step [75]. The NLRP3 activators induce mitochondrial damage, leading to PINK1-mediated Parkin recruitment to the damaged mitochondria, where Parkin ubiquitinates multiple proteins of the mitochondria outer membrane. p62 then recognizes ubiquitin-decorated mitochondria via its ubiquitin-associated (UBA) domain and delivers them to the autophagosome through interacting with LC3 via its LC3-interacting region (LIR) [3,75]. Our work, summarized in Figure 1, not only confirmed the fundamental role of the mitochondria in NLRP3 inflammasome activation, but also further established the “NF-κB−p62-mitophagy” axis as a macrophage-intrinsic negative regulatory mechanism that keeps the NLRP3 inflammasome activity in check to avoid immunopathology [75].

## 5. NLRP3 Inflammasome and Cancer

**Figure 2:**
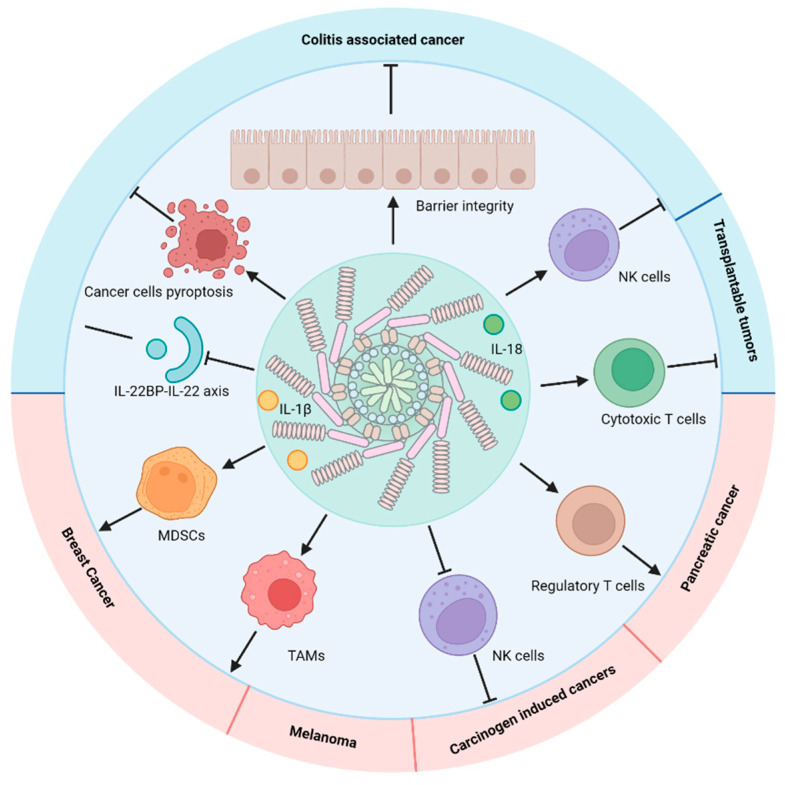
The NF-κB−NLRP3 inflammasome axis regulates tumorigenesis through modulating the tumor microenvironment. NLRP3 inflammasome-induced IL-1β production promotes MDSCs’ and TAMs’ infiltration, thereby driving breast cancer progression. NLRP3 could also suppress NK-cell and IFN-γ mediated antitumor responses in carcinogen-induced cancers and melanoma. Moreover, NLRP3 signaling drives pancreatic tumorigenesis by inducing tolerogenic T-cell differentiation and adaptive immune suppression. In contrast to these tumor-promoting effects, the NLRP3 inflammasome also suppresses tumorigenesis. For instance, NLRP3 inflammasome-dependent IL-1β production by dendritic cells directs an effective CD8^+^ T cell response against transplantable tumors. In colitis-associated colorectal cancer, NLRP3 inflammasome-induced IL-18 promotes an epithelial barrier healing process to prevent colorectal cancer progression and metastasis. Furthermore, IL-18 can also promote the tumoricidal activity of NK cells against metastasized colonic tumors and directly induce cancer cell pyroptosis. Lastly, NLRP3 inflammasome-dependent IL-18 downregulates the IL-22-binding protein (IL-22BP), whose production fine tunes IL-22 biological activity to regulate colonic tumorigenesis (the figure was created using BioRender).

As a dominant sensor for sterile inflammatory insults, the NLRP3 inflammasome, whose activity is orchestrated by NF-κB in the tumor microenvironment (TME), plays vital roles in regulating tumorigenesis (Figure 2) [79]. Although polymorphisms in NLRP3 inflammasome-related genes, including *NLRP3*, *CARD-8*, *IL-1β*, and *IL-18*, correlate with susceptibility, prognosis, and overall survival in different types of cancer [80,81,82,83,84,85,86], the precise function of the NLRP3 inflammasome in cancer appears to be context-dependent, as it can exert both anti- and pro-tumorigenic effects [87,88]. For instance, in breast cancer, NLRP3 inflammasome-induced IL-1β production promotes infiltration with immunosuppressive myeloid-derived suppressor cells (MDSCs) and tumor-associated macrophages (TAMs), generating a TME favoring breast cancer progression and metastasis [89,90,91,92]. Moreover, NLRP3 has also been shown to suppress NK cell and IFN-γ mediated antitumor responses to carcinogen-induced cancers in mice [93]. Additionally, the NLRP3 inflammasome is constitutively expressed and activated in human melanoma cells, promoting the secretion of IL-1β at late stages of the disease, to drive disease progression [94,95]. NLRP3 signaling also participates in pancreatic tumorigenesis by promoting tolerogenic T-cell differentiation and adaptive immune suppression via IL-10 [96]. Lastly, the NLRP3 inflammasome contributes to the development of myeloid leukemias, where its activation has been found in chronic myelomonocytic leukemia (CMML), juvenile myelomonocytic leukemia (JMML), and acute myeloid leukemia (AML) patients harboring *KRAS* mutations [97,98]. In further support of the pro-tumor role of the NLRP3 inflammasome, IL-1β neutralizing antibodies were recently found to attenuate lung cancer development in a large clinical trial (CANTOS) [99,100].

The NLRP3 inflammasome, however, also possesses anti-tumorigenic functions (Figure 2). For instance, NLRP3-dependent IL-1β production by dendritic cells is required for priming IFN-γ-producing T cells, and is thus essential for mounting an effective CD8^+^ T cell response against transplantable tumors [101,102]. In colitis-associated colorectal cancer, the NLRP3 inflammasome acts as a negative modulator of tumorigenesis [103], because NLRP3-dependent IL-18 production promotes epithelial barrier healing, thereby preventing colorectal cancer progression and metastasis [104,105,106,107,108,109]. Furthermore, IL-18 can also induce tumoricidal NK cell activity against metastasized colonic tumor cells in the mouse liver [105], and inflammasome-dependent pyroptosis in cancer cells exerts direct tumoricidal effects [103,110,111]. Lastly, NLRP3 inflammasome-dependent IL-18 downregulates IL-22-binding protein (IL-22BP), whose production orchestrates IL-22 biological activity, thereby suppressing intestinal damage at the peak of inflammation [112]. Altogether, these studies highlight the tumor suppressive role of the NLRP3 inflammasome. Further investigation of the interplay between NF-κB and the NLRP3 inflammasome should broaden our understanding about the complex roles of NF-κB and TME in tumorigenesis, and may also lead to the development of new anti-cancer therapies.

## 6. Conclusions

Inflammation is an evolutionarily conserved host protective mechanism whose activity requires precise calibration to ensure the rapid clearance of insults while avoiding immunopathology. NF-κB signaling represents a rapid and potent response to exogenous or endogenous insults and plays a central and pleiotropic role in shaping the outcome of inflammation, including regulating inflammasome activation and the efficacy of anti-cancer therapies. In addition to inducing the expression of pro-inflammatory cytokines, chemokines, and cell-survival factors, NF-κB also controls the expression of anti-inflammatory and antiapoptotic molecules that fine tune host immune responses [3,13,14,17]. Therefore, it seems oversimplified to define NF-κB as a pure pro- or anti-inflammatory transcription factor. Consistent with its complex biological functions, therapies that globally target NF-κB are impractical for the treatment of inflammation-associated disorders, including cancer. As NF-κB controls both the gas pedal and brake of inflammation, future investigations aiming at identifying NF-κB downstream factors and pathways that have clear pro- or anti-inflammatory roles should pave the way for developing new therapies to combat many inflammation-associated diseases, including cancer.

## Data Availability

Not applicable.

## References

[1] Zhong Z., Sanchez-Lopez E., Karin M. (2016). Autophagy, NLRP3 inflammasome and auto-inflammatory/immune diseases. Clin. Exp. Rheumatol..

[2] Sugimoto M.A., Sousa L.P., Pinho V., Perretti M., Teixeira M.M. (2016). Resolution of Inflammation: What Controls Its Onset?. Front. Immunol..

[3] Afonina I.S., Zhong Z., Karin M., Beyaert R. (2017). Limiting inflammation-the negative regulation of NF-kappaB and the NLRP3 inflammasome. Nat. Immunol..

[4] Lepelley A., Ghosh S. (2016). Clean Up after Yourself. Mol. Cell.

[5] Medzhitov R. (2008). Origin and physiological roles of inflammation. Nature.

[6] Martinon F., Mayor A., Tschopp J. (2009). The inflammasomes: Guardians of the body. Annu. Rev. Immunol..

[7] Gross O., Thomas C.J., Guarda G., Tschopp J. (2011). The inflammasome: An integrated view. Immunol. Rev..

[8] Zhong Z., Sanchez-Lopez E., Karin M. (2016). Autophagy, Inflammation, and Immunity: A Troika Governing Cancer and Its Treatment. Cell.

[9] Brydges S.D., Broderick L., McGeough M.D., Pena C.A., Mueller J.L., Hoffman H.M. (2013). Divergence of IL-1, IL-18, and cell death in NLRP3 inflammasomopathies. J. Clin. Investig..

[10] Guo H., Callaway J.B., Ting J.P. (2015). Inflammasomes: Mechanism of action, role in disease, and therapeutics. Nat. Med..

[11] Broderick L., De Nardo D., Franklin B.S., Hoffman H.M., Latz E. (2015). The inflammasomes and autoinflammatory syndromes. Annu. Rev. Pathol..

[12] Sen R., Baltimore D. (1986). Multiple nuclear factors interact with the immunoglobulin enhancer sequences. Cell.

[13] Zhang Q., Lenardo M.J., Baltimore D. (2017). 30 Years of NF-kappaB: A Blossoming of Relevance to Human Pathobiology. Cell.

[14] Taniguchi K., Karin M. (2018). NF-kappaB, inflammation, immunity and cancer: Coming of age. Nat. Rev. Immunol..

[15] Baltimore D. (2011). NF-kappaB is 25. Nat. Immunol..

[16] Ruland J. (2011). Return to homeostasis: Downregulation of NF-kappaB responses. Nat. Immunol..

[17] Ben-Neriah Y., Karin M. (2011). Inflammation meets cancer, with NF-kappaB as the matchmaker. Nat. Immunol..

[18] Greten F.R., Arkan M.C., Bollrath J., Hsu L.C., Goode J., Miething C., Goktuna S.I., Neuenhahn M., Fierer J., Paxian S. (2007). NF-kappaB is a negative regulator of IL-1beta secretion as revealed by genetic and pharmacological inhibition of IKKbeta. Cell.

[19] Perkins N.D. (2012). The diverse and complex roles of NF-kappaB subunits in cancer. Nat. Rev. Cancer.

[20] Smale S.T. (2012). Dimer-specific regulatory mechanisms within the NF-kappaB family of transcription factors. Immunol. Rev..

[21] Staudt L.M. (2010). Oncogenic activation of NF-kappaB. Cold Spring Harb. Perspect. Biol..

[22] Yu H., Lin L., Zhang Z., Zhang H., Hu H. (2020). Targeting NF-kappaB pathway for the therapy of diseases: Mechanism and clinical study. Signal Transduct. Target. Ther..

[23] Wang K., Kim M.K., Di Caro G., Wong J., Shalapour S., Wan J., Zhang W., Zhong Z., Sanchez-Lopez E., Wu L.W. (2014). Interleukin-17 receptor a signaling in transformed enterocytes promotes early colorectal tumorigenesis. Immunity.

[24] Schwitalla S., Ziegler P.K., Horst D., Becker V., Kerle I., Begus-Nahrmann Y., Lechel A., Rudolph K.L., Langer R., Slotta-Huspenina J. (2013). Loss of p53 in enterocytes generates an inflammatory microenvironment enabling invasion and lymph node metastasis of carcinogen-induced colorectal tumors. Cancer Cell.

[25] Sun S.C. (2017). The non-canonical NF-kappaB pathway in immunity and inflammation. Nat. Rev. Immunol..

[26] Senftleben U., Cao Y., Xiao G., Greten F.R., Krahn G., Bonizzi G., Chen Y., Hu Y., Fong A., Sun S.C. (2001). Activation by IKKalpha of a second, evolutionary conserved, NF-kappa B signaling pathway. Science.

[27] Smale S.T. (2011). Hierarchies of NF-kappaB target-gene regulation. Nat. Immunol..

[28] Kaser A., Zeissig S., Blumberg R.S. (2010). Inflammatory bowel disease. Annu. Rev. Immunol..

[29] Pasparakis M. (2009). Regulation of tissue homeostasis by NF-kappaB signalling: Implications for inflammatory diseases. Nat. Rev. Immunol..

[30] Bottini N., Firestein G.S. (2013). Duality of fibroblast-like synoviocytes in RA: Passive responders and imprinted aggressors. Nat. Rev. Rheumatol..

[31] Lizzul P.F., Aphale A., Malaviya R., Sun Y., Masud S., Dombrovskiy V., Gottlieb A.B. (2005). Differential expression of phosphorylated NF-kappaB/RelA in normal and psoriatic epidermis and downregulation of NF-kappaB in response to treatment with etanercept. J. Investig. Dermtol..

[32] Pasparakis M., Haase I., Nestle F.O. (2014). Mechanisms regulating skin immunity and inflammation. Nat. Rev. Immunol..

[33] Lowes M.A., Suarez-Farinas M., Krueger J.G. (2014). Immunology of psoriasis. Annu. Rev. Immunol..

[34] Neurath M.F., Pettersson S., Meyer zum Buschenfelde K.H., Strober W. (1996). Local administration of antisense phosphorothioate oligonucleotides to the p65 subunit of NF-kappa B abrogates established experimental colitis in mice. Nat. Med..

[35] MacMaster J.F., Dambach D.M., Lee D.B., Berry K.K., Qiu Y., Zusi F.C., Burke J.R. (2003). An inhibitor of IkappaB kinase, BMS-345541, blocks endothelial cell adhesion molecule expression and reduces the severity of dextran sulfate sodium-induced colitis in mice. Inflamm. Res..

[36] Gillooly K.M., Pattoli M.A., Taylor T.L., Chen L., Cheng L., Gregor K.R., Whitney G.S., Susulic V., Watterson S.H., Kempson J. (2009). Periodic, partial inhibition of IkappaB Kinase beta-mediated signaling yields therapeutic benefit in preclinical models of rheumatoid arthritis. J. Pharm. Exp..

[37] Schopf L., Savinainen A., Anderson K., Kujawa J., DuPont M., Silva M., Siebert E., Chandra S., Morgan J., Gangurde P. (2006). IKKbeta inhibition protects against bone and cartilage destruction in a rat model of rheumatoid arthritis. Arthritis Rheum..

[38] Tak P.P., Gerlag D.M., Aupperle K.R., Van De Geest D.A., Overbeek M., Bennett B.L., Boyle D.L., Manning A.M., Firestein G.S. (2001). Inhibitor of nuclear factor κB kinase β is a key regulator of synovial inflammation. Arthritis Rheum..

[39] Van Welden S., Selfridge A.C., Hindryckx P. (2017). Intestinal hypoxia and hypoxia-induced signalling as therapeutic targets for IBD. Nat. Rev. Gastroenterol. Hepatol..

[40] Rao P., Hayden M.S., Long M., Scott M.L., West A.P., Zhang D., Oeckinghaus A., Lynch C., Hoffmann A., Baltimore D. (2010). IkappaBbeta acts to inhibit and activate gene expression during the inflammatory response. Nature.

[41] Bohrer H., Qiu F., Zimmermann T., Zhang Y., Jllmer T., Mannel D., Bottiger B.W., Stern D.M., Waldherr R., Saeger H.D. (1997). Role of NFkappaB in the mortality of sepsis. J. Clin. Investig..

[42] McCall C.E., Yoza B.K. (2007). Gene silencing in severe systemic inflammation. Am. J. Respir. Crit. Care Med..

[43] Greten F.R., Eckmann L., Greten T.F., Park J.M., Li Z.W., Egan L.J., Kagnoff M.F., Karin M. (2004). IKKbeta links inflammation and tumorigenesis in a mouse model of colitis-associated cancer. Cell.

[44] Nenci A., Becker C., Wullaert A., Gareus R., van Loo G., Danese S., Huth M., Nikolaev A., Neufert C., Madison B. (2007). Epithelial NEMO links innate immunity to chronic intestinal inflammation. Nature.

[45] Hsu L.C., Enzler T., Seita J., Timmer A.M., Lee C.Y., Lai T.Y., Yu G.Y., Lai L.C., Temkin V., Sinzig U. (2011). IL-1beta-driven neutrophilia preserves antibacterial defense in the absence of the kinase IKKbeta. Nat. Immunol..

[46] Lawrance I.C., Wu F., Leite A.Z., Willis J., West G.A., Fiocchi C., Chakravarti S. (2003). A murine model of chronic inflammation-induced intestinal fibrosis down-regulated by antisense NF-kappa B. Gastroenterology.

[47] Segain J.P., Raingeard de la Bletiere D., Sauzeau V., Bourreille A., Hilaret G., Cario-Toumaniantz C., Pacaud P., Galmiche J.P., Loirand G. (2003). Rho kinase blockade prevents inflammation via nuclear factor kappa B inhibition: Evidence in Crohn’s disease and experimental colitis. Gastroenterology.

[48] Salim P.H., Xavier R.M. (2014). Influence of genetic polymorphisms (IL-10/CXCL8/CXCR2/NFkappaB) on the susceptibility of autoimmune rheumatic diseases. Rev. Bras. Reum..

[49] Li J., Tang R.S., Shi Z., Li J.Q. (2020). Nuclear factor-kappaB in rheumatoid arthritis. Int. J. Rheum. Dis..

[50] Canestri S., Totaro M.C., Serone E., Tolusso B., Frezza D., Gremese E., Ferraccioli G. (2012). Association between the response to B cell depletion therapy and the allele*2 of the HS1,2A enhancer in seropositive rheumatoid arthritis patients. Reumatismo.

[51] Luo J.L., Kamata H., Karin M. (2005). The anti-death machinery in IKK/NF-kappaB signaling. J. Clin. Immunol..

[52] Nenci A., Huth M., Funteh A., Schmidt-Supprian M., Bloch W., Metzger D., Chambon P., Rajewsky K., Krieg T., Haase I. (2006). Skin lesion development in a mouse model of incontinentia pigmenti is triggered by NEMO deficiency in epidermal keratinocytes and requires TNF signaling. Hum. Mol. Genet..

[53] Mangan M.S.J., Olhava E.J., Roush W.R., Seidel H.M., Glick G.D., Latz E. (2018). Targeting the NLRP3 inflammasome in inflammatory diseases. Nat. Rev. Drug Discov..

[54] Strowig T., Henao-Mejia J., Elinav E., Flavell R. (2012). Inflammasomes in health and disease. Nature.

[55] Muruve D.A., Petrilli V., Zaiss A.K., White L.R., Clark S.A., Ross P.J., Parks R.J., Tschopp J. (2008). The inflammasome recognizes cytosolic microbial and host DNA and triggers an innate immune response. Nature.

[56] Elliott E.I., Sutterwala F.S. (2015). Initiation and perpetuation of NLRP3 inflammasome activation and assembly. Immunol. Rev..

[57] Hennig P., Fenini G., Di Filippo M., Karakaya T., Beer H.D. (2021). The Pathways Underlying the Multiple Roles of p62 in Inflammation and Cancer. Biomedicines.

[58] Shi J., Zhao Y., Wang K., Shi X., Wang Y., Huang H., Zhuang Y., Cai T., Wang F., Shao F. (2015). Cleavage of GSDMD by inflammatory caspases determines pyroptotic cell death. Nature.

[59] McKee C.M., Coll R.C. (2020). NLRP3 inflammasome priming: A riddle wrapped in a mystery inside an enigma. J. Leukoc. Biol..

[60] Kelley N., Jeltema D., Duan Y., He Y. (2019). The NLRP3 Inflammasome: An Overview of Mechanisms of Activation and Regulation. Int. J. Mol. Sci..

[61] Swanson K.V., Deng M., Ting J.P. (2019). The NLRP3 inflammasome: Molecular activation and regulation to therapeutics. Nat. Rev. Immunol..

[62] Schroder K., Tschopp J. (2010). The inflammasomes. Cell.

[63] Bauernfeind F.G., Horvath G., Stutz A., Alnemri E.S., MacDonald K., Speert D., Fernandes-Alnemri T., Wu J., Monks B.G., Fitzgerald K.A. (2009). Cutting edge: NF-kappaB activating pattern recognition and cytokine receptors license NLRP3 inflammasome activation by regulating NLRP3 expression. J. Immunol..

[64] Franchi L., Eigenbrod T., Nunez G. (2009). Cutting edge: TNF-alpha mediates sensitization to ATP and silica via the NLRP3 inflammasome in the absence of microbial stimulation. J. Immunol..

[65] Juliana C., Fernandes-Alnemri T., Kang S., Farias A., Qin F., Alnemri E.S. (2012). Non-transcriptional priming and deubiquitination regulate NLRP3 inflammasome activation. J. Biol. Chem..

[66] Schroder K., Sagulenko V., Zamoshnikova A., Richards A.A., Cridland J.A., Irvine K.M., Stacey K.J., Sweet M.J. (2012). Acute lipopolysaccharide priming boosts inflammasome activation independently of inflammasome sensor induction. Immunobiology.

[67] Bezbradica J.S., Coll R.C., Schroder K. (2017). Sterile signals generate weaker and delayed macrophage NLRP3 inflammasome responses relative to microbial signals. Cell. Mol. Immunol..

[68] Fernandes-Alnemri T., Kang S., Anderson C., Sagara J., Fitzgerald K.A., Alnemri E.S. (2013). Cutting edge: TLR signaling licenses IRAK1 for rapid activation of the NLRP3 inflammasome. J. Immunol..

[69] Lin K.M., Hu W., Troutman T.D., Jennings M., Brewer T., Li X., Nanda S., Cohen P., Thomas J.A., Pasare C. (2014). IRAK-1 bypasses priming and directly links TLRs to rapid NLRP3 inflammasome activation. Proc. Natl. Acad. Sci. USA.

[70] Song N., Liu Z.S., Xue W., Bai Z.F., Wang Q.Y., Dai J., Liu X., Huang Y.J., Cai H., Zhan X.Y. (2017). NLRP3 Phosphorylation Is an Essential Priming Event for Inflammasome Activation. Mol. Cell.

[71] Shimada K., Crother T.R., Karlin J., Dagvadorj J., Chiba N., Chen S., Ramanujan V.K., Wolf A.J., Vergnes L., Ojcius D.M. (2012). Oxidized mitochondrial DNA activates the NLRP3 inflammasome during apoptosis. Immunity.

[72] Zhou R., Yazdi A.S., Menu P., Tschopp J. (2011). A role for mitochondria in NLRP3 inflammasome activation. Nature.

[73] Nakahira K., Haspel J.A., Rathinam V.A., Lee S.J., Dolinay T., Lam H.C., Englert J.A., Rabinovitch M., Cernadas M., Kim H.P. (2011). Autophagy proteins regulate innate immune responses by inhibiting the release of mitochondrial DNA mediated by the NALP3 inflammasome. Nat. Immunol..

[74] Medzhitov R. (2010). Inflammation 2010: New adventures of an old flame. Cell.

[75] Zhong Z., Umemura A., Sanchez-Lopez E., Liang S., Shalapour S., Wong J., He F., Boassa D., Perkins G., Ali S.R. (2016). NF-kappaB Restricts Inflammasome Activation via Elimination of Damaged Mitochondria. Cell.

[76] Liu T., Yamaguchi Y., Shirasaki Y., Shikada K., Yamagishi M., Hoshino K., Kaisho T., Takemoto K., Suzuki T., Kuranaga E. (2014). Single-cell imaging of caspase-1 dynamics reveals an all-or-none inflammasome signaling response. Cell Rep..

[77] Saitoh T., Fujita N., Jang M.H., Uematsu S., Yang B.G., Satoh T., Omori H., Noda T., Yamamoto N., Komatsu M. (2008). Loss of the autophagy protein Atg16L1 enhances endotoxin-induced IL-1beta production. Nature.

[78] Umemura A., He F., Taniguchi K., Nakagawa H., Yamachika S., Font-Burgada J., Zhong Z., Subramaniam S., Raghunandan S., Duran A. (2016). p62, Upregulated during Preneoplasia, Induces Hepatocellular Carcinogenesis by Maintaining Survival of Stressed HCC-Initiating Cells. Cancer Cell.

[79] Di Virgilio F. (2013). The therapeutic potential of modifying inflammasomes and NOD-like receptors. Pharm. Rev..

[80] Hamarsheh S., Zeiser R. (2020). NLRP3 Inflammasome Activation in Cancer: A Double-Edged Sword. Front. Immunol..

[81] Ungerback J., Belenki D., Jawad ul-Hassan A., Fredrikson M., Fransen K., Elander N., Verma D., Soderkvist P. (2012). Genetic variation and alterations of genes involved in NFkappaB/TNFAIP3- and NLRP3-inflammasome signaling affect susceptibility and outcome of colorectal cancer. Carcinogenesis.

[82] Verma D., Bivik C., Farahani E., Synnerstad I., Fredrikson M., Enerback C., Rosdahl I., Soderkvist P. (2012). Inflammasome polymorphisms confer susceptibility to sporadic malignant melanoma. Pigment Cell Melanoma Res..

[83] Miskiewicz A., Szparecki G., Durlik M., Rydzewska G., Ziobrowski I., Gorska R. (2015). The Q705K and F359L Single-Nucleotide Polymorphisms of NOD-Like Receptor Signaling Pathway: Association with Chronic Pancreatitis, Pancreatic Cancer, and Periodontitis. Arch. Immunol. Exp..

[84] Castano-Rodriguez N., Kaakoush N.O., Goh K.L., Fock K.M., Mitchell H.M. (2014). The NOD-like receptor signalling pathway in Helicobacter pylori infection and related gastric cancer: A case-control study and gene expression analyses. PLoS ONE.

[85] Zhang A., Yu J., Yan S., Zhao X., Chen C., Zhou Y., Zhao X., Hua M., Wang R., Zhang C. (2018). The genetic polymorphism and expression profiles of NLRP3 inflammasome in patients with chronic myeloid leukemia. Hum. Immunol..

[86] Wang H., Hua M., Wang S., Yu J., Chen C., Zhao X., Zhang C., Zhong C., Wang R., He N. (2017). Genetic polymorphisms of IL-18 rs1946518 and IL-1beta rs16944 are associated with prognosis and survival of acute myeloid leukemia. Inflamm. Res..

[87] Karki R., Kanneganti T.D. (2019). Diverging inflammasome signals in tumorigenesis and potential targeting. Nat. Rev. Cancer.

[88] Kantono M., Guo B. (2017). Inflammasomes and Cancer: The Dynamic Role of the Inflammasome in Tumor Development. Front. Immunol..

[89] Guo B., Fu S., Zhang J., Liu B., Li Z. (2016). Targeting inflammasome/IL-1 pathways for cancer immunotherapy. Sci. Rep..

[90] Ershaid N., Sharon Y., Doron H., Raz Y., Shani O., Cohen N., Monteran L., Leider-Trejo L., Ben-Shmuel A., Yassin M. (2019). NLRP3 inflammasome in fibroblasts links tissue damage with inflammation in breast cancer progression and metastasis. Nat. Commun..

[91] Kaplanov I., Carmi Y., Kornetsky R., Shemesh A., Shurin G.V., Shurin M.R., Dinarello C.A., Voronov E., Apte R.N. (2019). Blocking IL-1beta reverses the immunosuppression in mouse breast cancer and synergizes with anti-PD-1 for tumor abrogation. Proc. Natl. Acad. Sci. USA.

[92] Weichand B., Popp R., Dziumbla S., Mora J., Strack E., Elwakeel E., Frank A.C., Scholich K., Pierre S., Syed S.N. (2017). S1PR1 on tumor-associated macrophages promotes lymphangiogenesis and metastasis via NLRP3/IL-1beta. J. Exp. Med..

[93] Chow M.T., Sceneay J., Paget C., Wong C.S., Duret H., Tschopp J., Moller A., Smyth M.J. (2012). NLRP3 suppresses NK cell-mediated responses to carcinogen-induced tumors and metastases. Cancer Res..

[94] Okamoto M., Liu W., Luo Y., Tanaka A., Cai X., Norris D.A., Dinarello C.A., Fujita M. (2010). Constitutively active inflammasome in human melanoma cells mediating autoinflammation via caspase-1 processing and secretion of interleukin-1beta. J. Biol. Chem..

[95] Dunn J.H., Ellis L.Z., Fujita M. (2012). Inflammasomes as molecular mediators of inflammation and cancer: Potential role in melanoma. Cancer Lett..

[96] Daley D., Mani V.R., Mohan N., Akkad N., Pandian G., Savadkar S., Lee K.B., Torres-Hernandez A., Aykut B., Diskin B. (2017). NLRP3 signaling drives macrophage-induced adaptive immune suppression in pancreatic carcinoma. J. Exp. Med..

[97] Hamarsheh S., Osswald L., Saller B.S., Unger S., De Feo D., Vinnakota J.M., Konantz M., Uhl F.M., Becker H., Lubbert M. (2020). Oncogenic Kras(G12D) causes myeloproliferation via NLRP3 inflammasome activation. Nat. Commun..

[98] Ratajczak M.Z., Bujko K., Cymer M., Thapa A., Adamiak M., Ratajczak J., Abdel-Latif A.K., Kucia M. (2020). The Nlrp3 inflammasome as a “rising star” in studies of normal and malignant hematopoiesis. Leukemia.

[99] Ridker P.M., Everett B.M., Thuren T., MacFadyen J.G., Chang W.H., Ballantyne C., Fonseca F., Nicolau J., Koenig W., Anker S.D. (2017). Antiinflammatory Therapy with Canakinumab for Atherosclerotic Disease. N. Engl. J. Med..

[100] Ridker P.M., MacFadyen J.G., Thuren T., Everett B.M., Libby P., Glynn R.J., Ridker P., Lorenzatti A., Krum H., Varigos J. (2017). Effect of interleukin-1β inhibition with canakinumab on incident lung cancer in patients with atherosclerosis: Exploratory results from a randomised, double-blind, placebo-controlled trial. Lancet.

[101] Huang Y., Wang H., Hao Y., Lin H., Dong M., Ye J., Song L., Wang Y., Li Q., Shan B. (2020). Myeloid PTEN promotes chemotherapy-induced NLRP3-inflammasome activation and antitumour immunity. Nat. Cell Biol..

[102] Ghiringhelli F., Apetoh L., Tesniere A., Aymeric L., Ma Y., Ortiz C., Vermaelen K., Panaretakis T., Mignot G., Ullrich E. (2009). Activation of the NLRP3 inflammasome in dendritic cells induces IL-1beta-dependent adaptive immunity against tumors. Nat. Med..

[103] Allen I.C., TeKippe E.M., Woodford R.M., Uronis J.M., Holl E.K., Rogers A.B., Herfarth H.H., Jobin C., Ting J.P. (2010). The NLRP3 inflammasome functions as a negative regulator of tumorigenesis during colitis-associated cancer. J. Exp. Med..

[104] Zaki M.H., Boyd K.L., Vogel P., Kastan M.B., Lamkanfi M., Kanneganti T.D. (2010). The NLRP3 inflammasome protects against loss of epithelial integrity and mortality during experimental colitis. Immunity.

[105] Dupaul-Chicoine J., Arabzadeh A., Dagenais M., Douglas T., Champagne C., Morizot A., Rodrigue-Gervais I.G., Breton V., Colpitts S.L., Beauchemin N. (2015). The Nlrp3 Inflammasome Suppresses Colorectal Cancer Metastatic Growth in the Liver by Promoting Natural Killer Cell Tumoricidal Activity. Immunity.

[106] Zaki M.H., Vogel P., Body-Malapel M., Lamkanfi M., Kanneganti T.D. (2010). IL-18 production downstream of the Nlrp3 inflammasome confers protection against colorectal tumor formation. J. Immunol..

[107] Dupaul-Chicoine J., Yeretssian G., Doiron K., Bergstrom K.S., McIntire C.R., LeBlanc P.M., Meunier C., Turbide C., Gros P., Beauchemin N. (2010). Control of intestinal homeostasis, colitis, and colitis-associated colorectal cancer by the inflammatory caspases. Immunity.

[108] Salcedo R., Worschech A., Cardone M., Jones Y., Gyulai Z., Dai R.M., Wang E., Ma W., Haines D., O’HUigin C. (2010). MyD88-mediated signaling prevents development of adenocarcinomas of the colon: Role of interleukin 18. J. Exp. Med..

[109] Takagi H., Kanai T., Okazawa A., Kishi Y., Sato T., Takaishi H., Inoue N., Ogata H., Iwao Y., Hoshino K. (2003). Contrasting action of IL-12 and IL-18 in the development of dextran sodium sulphate colitis in mice. Scand. J. Gastroenterol..

[110] Zhong Z., Zhai Y., Bu P., Shah S., Qiao L. (2017). Papilloma-pseudovirus eradicates intestinal tumours and triples the lifespan of Apc(Min/+) mice. Nat. Commun..

[111] Wang Y., Gao W., Shi X., Ding J., Liu W., He H., Wang K., Shao F. (2017). Chemotherapy drugs induce pyroptosis through caspase-3 cleavage of a gasdermin. Nature.

[112] Huber S., Gagliani N., Zenewicz L.A., Huber F.J., Bosurgi L., Hu B., Hedl M., Zhang W., O’Connor W., Murphy A.J. (2012). IL-22BP is regulated by the inflammasome and modulates tumorigenesis in the intestine. Nature.

